# Disulfiram Efficacy in the Treatment of Alcohol Dependence: A Meta-Analysis

**DOI:** 10.1371/journal.pone.0087366

**Published:** 2014-02-10

**Authors:** Marilyn D. Skinner, Pierre Lahmek, Héloïse Pham, Henri-Jean Aubin

**Affiliations:** 1 Centre de Traitement des Addictions, Hôpital Emile Roux, Assistance Publique-Hôpitaux de Paris, Institut National de la Santé et de la Recherche Médicale U669, Limeil-Brévannes, France; 2 Centre de Traitement des Addictions, Hôpital Emile Roux, Assistance Publique-Hôpitaux de Paris, Limeil-Brévannes, France; 3 Centre Médical Marmottan Paris, France; 4 Centre d'Enseignement, de Recherche et de Traitement des Addictions, Hôpital Paul Brousse, Assistance Publique-Hôpitaux de Paris, Université Paris-Sud, Institut National de la Santé et de la Recherche Médicale U669, Villejuif, France; Federal University of Rio de Janeiro, Brazil

## Abstract

**Background:**

Despite its success with compliant or supervised patients, disulfiram has been a controversial medication in the treatment of alcoholism. Often, study designs did not recognize a pivotal factor in disulfiram research, the importance of an open-label design. Our objectives are: (1) to analyze the efficacy and safety of disulfiram in RCTs in supporting abstinence and (2) to compare blind versus open-label studies, hypothesizing that blinded studies would show no difference between disulfiram and control groups because the threat would be evenly spread across all groups.

**Methods and Findings:**

We searched PubMed, EMBASE and the Cochrane Central Register for RCTs on disulfiram use with alcoholics in comparison to any alcoholic control group. The primary outcome was defined by the authors of each trial. Additional analyses included: blind vs. open-label, with or without supervision, cocaine study or not, and type of control.

Overall, the 22 included studies showed a higher success rate of disulfiram compared to controls Hedges'g = .58 (95%CI = .35–.82). When comparing blind and open-label RCTs, only open-label trials showed a significant superiority over controls g = .70 (95%CI = .46–.93). RCTs with blind designs showed no efficacy of disulfiram compared to controls. Disulfiram was also more effective than the control condition when compared to naltrexone g = .77, 95%CI = .52–1.02, to acamprosate g = .76, 95%CI = .04–1.48, and to the no disulfiram groups g = .43, 95%CI = .17–.69.

Limits include: (1) a population of 89% male subjects and (2) a high but unavoidable heterogeneity of the studies with a substantial I-square in most subgroups of studies.

**Conclusions:**

Blinded studies were incapable of distinguishing a difference between treatment groups and thus are incompatible with disulfiram research. Based on results with open-label studies, disulfiram is a safe and efficacious treatment compared to other abstinence supportive pharmacological treatments or to no disulfiram in supervised studies for problems of alcohol abuse or dependence.

## Introduction

Disulfiram has been used in the treatment of alcohol dependence with consistently successful results in individuals with high compliance or when medication intake has been directly supervised [1], [2], [3]. Its mechanism of action for maintaining alcohol abstinence is thought to be primarily psychological [4], [5], [6], [7] and based on a highly disagreeable pharmacological effect if alcohol is consumed. Disulfiram blocks the enzyme aldehyde dehydrogenase (ALDH). If alcohol is present, acetaldehyde accumulates [8], [9] usually resulting in an unpleasant reaction, the disulfiram-ethanol reaction (DER), consisting primarily of tachycardia, flushing, nausea, and vomiting [8]. To prevent the first drink, however, the psychological or cognitive threat is thought to be dominant and active and thus dissuade use [5], [6], [7]. The threat of a DER, indeed the expectancy of negative consequences if alcohol were to be absorbed and ensuing thoughts about avoiding pain and sickness account for the drug's effectiveness.

On the other hand, different pharmacodynamic rather than psychological mechanisms of action have been proposed to explain the success of disulfiram in cocaine addiction [9], [10], [11], and in one case report of pathological gambling [12]. Several studies have proposed that cocaine use is reduced in subjects taking disulfiram because disulfiram inhibits dopamine beta-hydroxylase (DBH) and the consequent reduction of synaptic norepinephrine release alters the “high” [13], [14], [15], [16].

Despite its apparent success with compliant or supervised alcohol dependent patients, efficacy studies of disulfiram have been all but concordant, leading to confusion and debates that are largely based on poorly designed studies. The principal methodological design flaws are failure to monitor compliance, absence of control groups, unmatched control groups, no clear objective measure of abstinence, and the problem of blinding in disulfiram studies [2], [17], [18]. The use of double-blind studies has long been considered the standard method of determining drug efficacy. The principal advantages of the blinding procedure are to minimize the effects of disulfiram biases such as perceptions and expectations arising from both patients and researchers about the drug's effects. Disulfiram, however, unlike other medications, is problematic in blind trials for two main reasons. First, the expectation of a DER in the disulfiram treated patients is thought to be directly related to the efficacy of the drug and thus the disulfiram group must be open to their treatment in order to maintain these expectancies. Likewise, the control group(s) must be open to their treatment in order to be free of the DER expectancy. Secondly, participants in a blinded trial can easily unmask the blind by taking a small dose of alcohol, leading to compliance problems. To summarize, the fundamental problem in blinded trials is that the psychological threat of a DER is present and active in both arms of a given trial, impeding a clear distinction between the active and placebo groups. Indeed, if we postulate that the psychological threat of an aversive reaction is the pivotal mechanism of action of the drug as opposed to its actual pharmacodynamic properties when combined with alcohol, then there would be no chance of finding any difference between disulfiram and a control group in a double-blind design [18].

To date, one meta-analysis specifically on disulfiram has been conducted on disulfiram efficacy for the treatment of alcohol use disorder [19]. Of eleven total studies, ten were selected and divided into five forest plots. Not included were trials with co-dependent alcohol and cocaine subjects and trials that combined disulfiram treatment with another treatment (such as a placebo or methadone). They concluded that supervised disulfiram had some effect on short-term abstinence, number of drinking days, and days until relapse compared to placebo, no disulfiram, or other treatments.

While providing results with useful implications, the Jorgensen et al analysis contained a number of shortcomings regarding content and design issues. Although Jorgensen et al proposed a meta-analysis on disulfiram efficacy, one non-efficacy study was included [20] and five eligible trials were absent [21], [22], [23], [24], [25]. Furthermore, the critical and determinant factor in disulfiram efficacy – that only open-label trials can show efficacy – was not explored by Jorgensen et al, and they mixed open and blind studies in their meta-analysis.

Our aim is to quantitatively demonstrate that disulfiram treatment is more effective in open-label rather than in blinded experiments, because in the later the psychological effect of the fear of a DER would be expected to have the same effect in both arms of the study. In addition, we expect that this analysis will show disulfiram to be more effective in supervised studies compared to unsupervised studies. Compliance has been a serious impediment in disulfiram research in numerous studies [26], [27].

## Methods

### Search strategy and study selection

We searched all controlled trials on disulfiram use with alcohol dependent patients using the PubMed database (last search date June 2012), EMBASE (last search date July 2012), the Cochrane Central Register (last search November 2012), and a manual search. The details of the search strategies can be found in Search Strategies S1.

Three investigators (MS, PL, HJA) independently read the abstracts in order to select the publications of interest. Included in this study were original, randomized controlled trials (RCTs) comparing the efficacy of orally administered disulfiram to any control group. All studies included subjects with a diagnosis of alcohol abuse or dependence, whatever the classification system. In some studies, the primary inclusion criterion was cocaine dependence. In this case, we performed the analysis in the comorbid alcohol-cocaine subset. These studies consisted of blind and open-label designs, both supervised and unsupervised.

### Data extraction

The data extraction was performed by two independent investigators (MS, HJA). Discrepancies were confronted and a consensus was agreed upon. When possible, authors were contacted by e-mail or personally to retrieve missing data, with a special emphasis on the primary endpoint.

We extracted the primary outcome as defined by the authors in their articles (Table 1). Additional variables were extracted for subgroup analysis: blind or open-label, with or without supervision, cocaine study or not, and type of control. We also included meta-regressions to explain heterogeneity (treatment duration, disulfiram dosage, and publication year, with a risk of bias score) [28].

**Table 1:** Study Description.

Study	Participants (Gender), Age (mean)	Principal inclusion criteria	Treatment duration	Intervention Arms (n)	Blind or Open	Super-vision	Particularity	Primary Outcome
Bardeleben et al 1999	60 (44♂), 44.8	Alcohol dependence (DSM-IV criteria)	12 weeks	1) DIS 200 mg/d (20) 2) ACA 1800 mg/d (20) 3) NTX 45 mg/d (20)	Open	Yes		% of abstinent days to treatment days
Carroll et al 1993	18 (13♂), 32	Alcohol dependence or abuse and cocaine dependence (DSM-III-R criteria)	12 weeks	1) DIS 250 mg/d (9) 2) NTX 50 mg/d (9)	Open	Yes		Mean days of alcohol use
Carroll et al 1998	122 (33♀), 30.8	Alcohol dependence or abuse and cocaine dependence (DSM-III-R criteria)	12 weeks	1) DIS 262 mg/d (mean) (78) 2) No DIS (44)	Open	Yes	Combination of arms	3 or more weeks consecutive abstinence
Carroll et al 2004	121 (63 analyzed who were co-dependent, gender & age not available in this subgroup)	Alcohol and cocaine dependence(DSM-IV criteria)	12 weeks	1) DIS 250 mg/d (38)2) Placebo (25)	Blind	No	Combination of arms	3 or more weeks consecutive abstinence
Chick et al 1992	126 (20♀), 43.0	Alcohol dependence (SADQ*)	26 weeks	1) DIS 200 mg/d (64)2) No DIS [Vit C 100 mg/d] (62)	Open	Yes		Days abstinent during last 6 months
De Sousa & De Sousa 2004	100 ♂, 44.4	Alcohol dependence (DSM-IV criteria)	52 weeks	1) DIS 250 mg/d (50) 2) NTX 50 mg/d (50)	Open	Yes		No relapse
De Sousa & De Sousa 2005	100 ♂, 42.2	Alcohol dependence (DSM-IV criteria)	34 weeks	1) DIS 250 mg/d (50) 2) ACA 1998 mg/d (50)	Open	Yes		No relapse
De Sousa et al 2008	100 ♂, 43.3	Alcohol dependence (DSM-IV criteria)	39 weeks	1) DIS 250 mg/d (50)2) TPM 150 mg/d (50)	Open	Yes		No relapse
De Sousa & De Sousa 2008	58 ♂, 17.3	Alcohol dependence (DSM-IV criteria)	26 weeks	1) DIS 250 mg/d (29) 2) NTX 50 mg/d (29)	Open	Yes		Total abstinence
De Sousa & Jagtap 2009	32 ♂, 66.1	Alcohol dependence (DSM-IV criteria)	26 weeks	1) DIS 250 mg/d (16) 2) NTX 100 mg/d (16)	Open	Yes		No relapse
Fuller & Roth 1979	128 ♂, 42.6	Alcohol dependence (No criteria cited)	52 weeks	1) DIS 250 mg/d (43)2) DIS 1 mg/d (43)3) No DIS [riboflavin 50 mg/d] (42)	Blind for comparisons 1 & 2, Open for comparisons 1 & 3	No	3 arms	Continuous abstinence
Fuller et al 1986	605 ♂, 41.7	Alcohol dependence (National Council on Alcoholism criteria)	52 weeks	1) DIS 250 mg/d (202)2) DIS 1 mg/d (204)3) No DIS [riboflavin 50 mg/d] (199)	Blind for comparisons 1 & 2,Open for comparisons 1 & 3	No	3 arms	Continuous abstinence
Gerrein et al 1973	121 (49 analyzed for this meta-analysis, gender & age not available in this subgroup)	Alcohol dependence (No criteria cited)	8 weeks	1) DIS 250 mg/d (26)2) No DIS (23)	Open	Yes (n = 13) No (n = 13)	6 arms	Total abstinence
Grassi et al 2007	12 ♂, 32.87	Alcohol and cocaine dependence (SDS **) criteria	12 weeks	1) DIS 400 mg/d (4)2) No DIS (4)3) NTX 50 mg/d (4)	Open	No	3 arms	4 weeks abstinence
Laaksonen et al 2008	243 (71♀), 43.1	Alcohol dependence (ICD-10 criteria)	12 weeks	1) DIS 150 mg/d (mean) (81)2) NTX 50 mg/d (81)3) ACA 1333-1998 mg/d (81)	Open	Yes	3 arms	Time to first heavy drinking day
Ling et al 1983	82 (gender not given), 39.0	Alcohol dependence or abuse (criteria not given)	36 weeks	1) DIS 250 mg/d (41)2) placebo (41)	Blind	Yes for subjects at the clinic.No for those with take home privileges	Methadone patients	No relapse and continued clinic attendance
Nava et al 2006	86 (18♀), 40.0	Alcohol dependence (DSM-IV criteria)	52 weeks	1) DIS 200 mg/d (31)2) NTX 50 mg/d (27)3) GHB 50 mg/kg of body weight/d (28)	Open	Yes	3 arms	No relapse
Niederhofer & Staffen 2003	49 (26 analyzed, 9♀), 17.0	Alcohol dependence, chronic or episodic (DSM-IV criteria)	12 weeks	1) DIS 200 mg/d (13)2) placebo (13)	Blind	No	Adolescents	Total abstinence
Petrakis et al 2000	67 (17 analyzed who were co-dependant, gender & age not available)	Alcohol dependence or abuse and cocaine dependence (DSM-IV criteria or psychiatric evaluation)	12 weeks	1) DIS 250 mg/d (8)2) placebo (9)	Blind	No	Methadone (disulfiram in methadone)	Total abstinence
Petrakis et al 2005	254 (189 analyzed, 5 ♀), 47.0	Alcohol dependence (DSM-IV criteria)	12 weeks	1) DIS 250 mg/d (66) + Placebo2) No DIS (64)3) NTX 50 mg/d (59)	Open	No	Combination of arms	Total abstinence
Pettinati et al 2008	208 (159 analyzed, 46 ♀), 41.0	Alcohol and cocaine dependence (DSM-IV criteria)	11 weeks	1) DIS 250 mg/d (53)2) Placebo (54)3) NTX 100 mg/d (52)	Blind	No	Combination of arms	At least 3 consecutive weeks of abstinence from both alcohol and cocaine
Ulrichsen et al 2010	39 (12♀), 52.0	Alcohol dependence(ICD-10 criteria)	26 weeks	1) DIS 176 mg/d (mean) (19)2) No DIS (20)	Open	Yes		Total abstinence

ACA Acamprosate, DIS disulfiram, NTX Naltrexone, TPM Topimirate, GHB *g*-hydroxybutyrate* SADQ Severity of Alcohol Dependence Questionnaire [66]** SDS- Severity of Dependence Scale questionnaire for cocaine and alcohol dependence.

### Study quality analysis

The methodological quality of the studies was analyzed according to the Cochrane Collaboration's tool for assessing risk of bias. The 6-item tool assesses the quality of the randomization procedure (adequate sequence generation and allocation concealment), the blinding of treatments, the probability of other bias, the probability of selective reporting, and issues of incomplete data.

### Data analysis

Efficacy outcomes were analyzed by calculating the Hedge's g effect-size for each trial with the uncertainty of each result being expressed by their 95% confidence intervals (CI). An effect-size of 0.2 to 0.3 is thought to be a “small” effect, around 0.5 a “medium” effect, and 0.8 to infinity, a “large” effect [28].

Effects were summarized using a random-effects model. The random-effects model was chosen because of the high heterogeneity between trial estimates in each meta-analysis. The underlying assumption of a random-effects model is that the true effect could vary between studies based on characteristics of the study population or intervention. In this case, for example, the primary outcomes may differ as a function of alcohol use disorder severity, intervention duration, comparator type, and associated psychosocial intervention intensity. A random-effect model is more conservative in that it produces wider confidence intervals for the effect estimates than a fixed-effects model.

Initially all studies were included in the analysis in order to have a global assessment of disulfiram efficacy, regardless of blind or open-label design. The second step was to test the hypothesis that disulfiram shows no difference compared to the control condition if its administration is blinded. Validating this hypothesis would signify that only open-label designs could indicate a treatment effect. In this case, the following analyses should be conducted only in the open-label condition: supervised versus non supervised administration, cocaine versus non cocaine studies, and disulfiram versus the various control conditions.

Publication bias was assessed using funnel plots. Heterogeneity was assessed by calculating the *I^2^* value. *I^2^* (range = 0–100%) quantifies the degree of variability, with cut-offs suggested as 0% to 40% (probably unimportant heterogeneity), 30% to 60% (moderate heterogeneity), 50% to 90% (substantial heterogeneity), and 75% to 100% (considerable heterogeneity). The importance of the observed value of I^2^ depends on the magnitude and direction of effects and the strength of evidence for heterogeneity (e.g., p-value from the chi-squared test, or a confidence interval for I^2^) [29]. Leave-one-out sensitivity analyses were conducted. Sources of bias and heterogeneity were evaluated using meta-regression (for publication year, study quality, treatment duration, and disulfiram dosage). A significance level of p<.05 (2-tailed) was used for all analyses.

The forest plot on safety was calculated using the number of events per person years.

All analyses were conducted using the Comprehensive Meta-Analysis statistical program, version 2.2.50 (Englewood, NJ).

## Results

### Included studies

**Figure 1:**
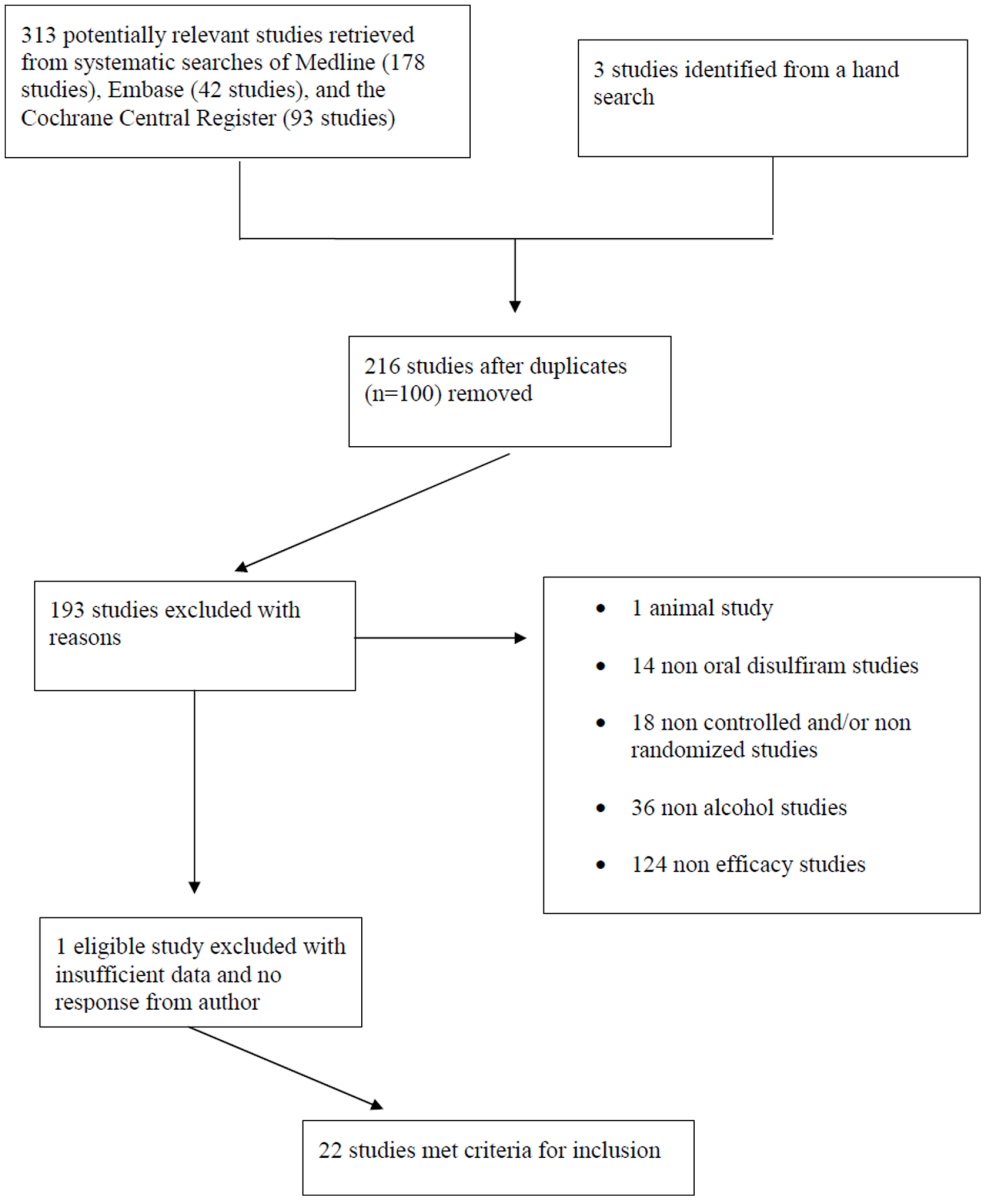
Flowchart of selection of studies for inclusion in the meta-analysis.

Systematic searches resulted in 178 references from PubMed, 42 from Embase, and 93 from the Cochrane Central Register. Of these 313 references, 100 were duplicates. A manual search resulted in 3 additional articles bringing the total to 216. After cross-referencing between investigators, 35 were then selected for a detailed review and analysis. From these, 23 were chosen for inclusion in the meta-analysis. In five of these studies, additional data were needed on the primary outcome of a subset of the participants. We obtained additional data from the authors in four of these studies. Because we did not obtain a response from the fifth author [30], we were unable to include their trial. The final selection procedure thus allowed us to analyze 22 studies (Figure 1).

### Description of the selected studies

The studies selected for this meta-analysis (Table 1) were published between 1973 and 2010. Most were from the United States (10), followed by India (5), Italy (2), and one each from the United Kingdom, Finland, Austria, Denmark, and Switzerland. The cumulative total of subjects analyzed in the 22 studies was 2414, ranging from 12 [13] to 605 [27]. Over half included 100 or more subjects. The subjects were alcohol dependent in 18 of the studies and diagnosed as alcohol abuse or dependent in the other four studies [14], [22], [31], [32]. For the studies indicating gender, 2058 were men and 266 were women (11.4%). The breakdown by gender was not available in a sub sample of two studies in which we analyzed only the arms of the study that were related to our research question [14], [25]. In one study, gender was not indicated [32]. Two studies evaluated adolescents (mean age 17) [33], [34], one evaluated an older population (>60 years old) [24]. The remaining studies analyzed subjects with a mean age range of 31–52 years.

Six of the studies evaluated a population of cocaine dependent subjects, of which all or a part were also alcohol dependent or abused alcohol [10], [13], [14], [21], [26], [31]. Where necessary, we extracted for analysis only the co-dependent subjects in these cocaine studies [10], [14].

In most studies, subjects were recruited from a center in which they were undergoing treatment for alcohol dependence [22], [23], [24], [33], [35], [36], [37], [38], [39] or seeking treatment and recruited through the media [10], [26], [31], [34], [40], [41]. The largest study's subjects (n = 605) were patients participating in VA (Veterans Administration) medical centers [27]. One study included patients admitted to a psychiatric center emergency ward [42], while three others recruited outpatients in alcohol or substance abuse clinics [13], [21], [25]. Finally in two studies, the subjects were either currently enrolled in a methadone clinic or had been discharged from one because of problems related to drinking [14], [32].

Only two of the 22 studies did not require counseling [32], [34]. In 20 studies patients generally received weekly group cognitive behavior therapy or less formal alcohol counseling.

Twelve studies required medication intake supervision by a family member, friend, or a member of the clinic staff while eight did not require this supervision. In the remaining two studies, half of the subjects were supervised in one case [25] and in the other, the study medication was to be taken with methadone and was supervised only in those without take home privileges [32]. In ten of the supervised studies, the control arm was also supervised when it consisted of another medication or a placebo. In the three remaining studies, the control arm was “no disulfiram” and thus no supervision was possible [25], [31], [42].

The most frequent measure of baseline alcohol consumption was the number of drinks consumed per drinking day. In the studies using this criterion, the range was 5–19 drinks with most studies reporting 8–13 drinks per day. The baseline alcohol consumption was not available in four studies [22], [34], [39], [42]. We were unable to obtain the baseline alcohol consumption for the sub sample of codependent patients in the Carroll et al study (2004) [43]. In three of the studies, other consumption measures were provided when the number of drinks per day was not stated: Fuller et al (1986) reported days drank in the prior month (20–21), Gerrein et al (1973) reported the number of years of loss of control drinking (12.83), and Ling et al (1982) reported that 80% of their sample drank daily.

The designs of the studies ranged from two to six arms. Eleven studies contained two arms comparing disulfiram to either placebo [14], [32], [34], to no disulfiram [23], [42], to naltrexone [21], [24], [33], [35], to acamprosate [36], or to topiramate [37]. Six studies had three arms. Four of these compared disulfiram to naltrexone and to one other condition: to no disulfiram [13], to acamprosate [22], [40], or to GHB [41]. The control arms in these studies were combined in order to calculate the overall efficacy and the following comparisons: blind versus open-label, supervision versus no supervision, and cocaine versus non cocaine. The control arms were analyzed separately, however, when comparing disulfiram efficacy across the various control conditions. The fifth and sixth studies compared disulfiram to placebo and no disulfiram [27], [39]. In these two studies, the control arms were combined in order to calculate the overall efficacy effect-size. In comparisons of efficacy in blind versus open-label, the control arms were analyzed separately. In addition, for comparisons of cocaine versus non cocaine trials, supervised versus non supervised conditions, and across the various control conditions, only the no disulfiram arm was analyzed to determine disulfiram efficacy and this part of both trials was open-label. When appropriate, arms were combined according to Higgins and Green [29].

In the Carroll et al (2004) four arm study in which two types of psychotherapy were combined with disulfiram or placebo in a blind design, we extracted only the patients who were alcohol dependent from the disulfiram and placebo groups regardless of their psychotherapy group allocation to form one comparison. In the Carroll et al (1998) five arm open study, also comparing different types of psychotherapy with disulfiram or with no disulfiram, we again extracted only the patients who were alcohol dependent from the combined disulfiram and no disulfiram groups.

The Gerrein et al (1973) six arm study compared unsupervised and supervised disulfiram intake (groups one and two) to two no disulfiram groups (groups three and four depending upon the frequency of clinic visits). Groups one and three were assigned weekly clinic visits and groups two and four biweekly clinic visits. The last two arms were not randomized and thus not included in our analyses. We compared the supervised (biweekly clinic visit) disulfiram group to the no disulfiram (also biweekly clinic visit) group and the unsupervised (weekly clinic visit) disulfiram group to the other no disulfiram (weekly clinic visit) group.

In the four arm study by Petrakis et al (2005) in which open-label disulfiram + blinded placebo were compared to placebo, naltrexone, or open-label disulfiram + blinded naltrexone, we analyzed two comparisons: disulfiram + placebo compared to naltrexone or compared to placebo. Because the placebos in this study were for naltrexone, we labeled this comparison disulfiram vs “no disulfiram” in our analysis. We did not analyze the comparisons in which disulfiram and naltrexone were administered together.

In the Pettinati et al (2008) double-blind, four arm study, similar comparisons were made. We analyzed disulfiram when administered alone, not with naltrexone. The placebo was double matched for both naltrexone and disulfiram. We analyzed disulfiram compared to naltrexone and the double placebo.

Fifteen of the studies were open-label, five were double-blind, and two contained a blind and an open-label design with the blind arm comparing disulfiram to placebo and the open arm comparing disulfiram to no disulfiram [27], [39].

Medication compliance was monitored by self-report (14 studies), friend or family member report (seven studies), a riboflavin compliance procedure to monitor self-report (three studies), pill count (three studies), linking disulfiram to methadone intake (two studies), using Microelective Events Monitoring (MEM) caps (one study), and supervision (thirteen studies). One study provided weekly clinical management compliance enhancement therapy [38].

The methodological quality of the studies was analyzed according to the Cochrane Collaboration's tool for assessing risk of bias [44]. The 6-item tool assesses the quality of the randomization procedure (adequate sequence generation and allocation concealment), the blinding of treatments, the probability of other bias, the probability of selective reporting, and the issue of incomplete data. When the studies were described as open-label studies, the blinding item was considered as non applicable. With this tool, a high score indicates a low risk of bias.

Among the 22 studies included, two met four of these six criteria, two met three criteria, eleven studies met two criteria, one met one criterion, and six studies met none of them (Table 2).

**Table 2:** Methodological Quality of Included Studies.

	Adequate sequence generation	Allocation concealment	Blinding of participants, personnel and outcome assessors	Free of other bias	Free of selective reporting	Incomplete outcome data addressed	Quality assessment(maximum score: 6/6)
**Bardeleben et al 1999**	?	-	NA	-	?	?	0
**Carroll et al 1993**	?	-	NA	-	?	?	0
**Carroll et al 1998**	?	?	?	-	?	-	0
**Carroll et al 2004**	+	?	+	+	?	+	4
**Chick et al 1992**	+	+	-	?	?	+	3
**De Sousa & De Sousa 2004**	+	?	NA	-	?	+	2
**De Sousa & De Sousa 2005**	+	?	NA	-	?	+	2
**De Sousa et al 2008**	+	?	NA	-	?	+	2
**De Sousa & De Sousa 2008**	+	-	NA	-	?	+	2
**De Sousa & Jagtap 2009**	+	-	NA	-	?	+	2
**Fuller & Roth 1979**	+	?	+	-	?	?	2
**Fuller et al 1986**	+	+	-	?	?	?	2
**Gerrein et al 1973**	?	?	NA	?	?	?	0
**Grassi et al 2007**	?	?	NA	-	-	-	0
**Laaksonen et al 2008**	+	?	NA	+	+	+	4
**Ling et al 1983**	?	?	+	?	-	-	1
**Nava et al 2006**	+	?	NA	?	?	+	2
**Niederhofer & Staffen 2003**	?	+	+	?	?	?	2
**Petrakis et al 2005**	?	?	NA	-	?	-	0
**Petrakis et al 2000**	?	?	+	-	+	+	3
**Pettinati et al 2008**	?	?	+	?	?	+	2
**Ulrichsen et al 2010**	+	-	NA	-	?	+	2

### Primary endpoint

The primary endpoint of this meta-analysis was the effect-size at the end of treatment for the key variable as defined by the authors in their articles. The treatment duration varied from 8 to 52 weeks. The effect-size variable could be: total abstinence, proportion of abstinent days to treatment days, mean days of alcohol use, no relapse, time to first heavy drinking day, or three or more weeks of consecutive abstinence as shown in Table 1. In most cocaine studies, the time point for the effect-size variable was earlier than the total study duration, as previous trials have shown that as little as three consecutive weeks of abstinence from both cocaine and alcohol is predictive of long-term cocaine abstinence [26]. Primary outcome success rates are shown in Table 3.

**Table 3:** Success Rates on Primary Outcomes.

Study Mean (SD) or %	Disulfiram success Rate	Disulfiram N	Control success rate	Control N
Bardeleben et al 1999	93.3 (16.6)	20	89.6 (18.04)	40
Carroll et al 1993	2.4 (2.3)	9	10.4 (7.7)	9
Carroll et al 1998	53.00%	78	16.00%	44
Carroll et al 2004	87.50%	38	82.60%	25
Chick et al 1992	100 (70)	47	69 (67)	46
De Sousa & De Sousa 2004	82.00%	50	42.00%	50
De Sousa & De Sousa 2005	88.00%	50	46.00%	50
De Sousa et al 2008	90.00%	50	56.00%	50
De Sousa & De Sousa 2008	79.31%	29	51.72%	29
De Sousa & Jagtap 2009	81.25%	16	43.75%	16
Fuller & Roth 1979	21.00%	43	18.58%	85
Fuller et al 1986	18.80%	202	19.34%	403
Gerrein et al 1973	23.07%	26	8.70%	23
Grassi et al 2007	100.00%	4	12.50%	8
Laaksonen et al 2008	46.6 (27.5)	33	17,87 (21,03)	91
Ling et al 1983	9.80%	41	24.40%	41
Nava et al 2006	90.00%	31	80.11%	55
Niederhofer & Staffen 2003	53.80%	13	15.40%	13
Petrakis et al 2000	100.00%	8	57.00%	9
Petrakis et al 2005	77.30%	66	65.02	123
Pettinati et al 2008	17.00%	53	16.13%	106
Ulrichsen et al 2010	26.00%	19	20.00%	20

### Disulfiram efficacy

**Figure 2:**
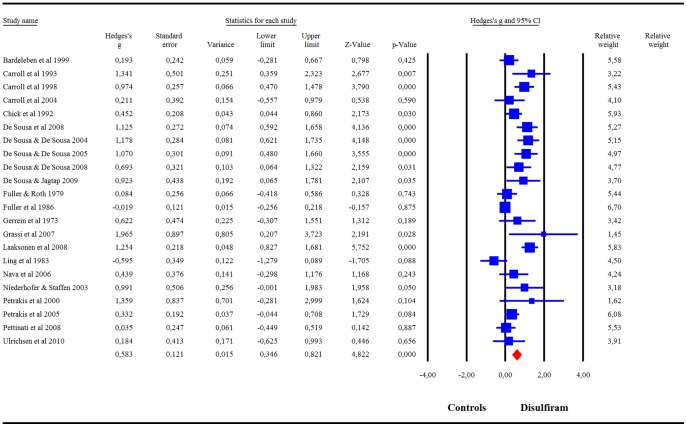
Meta-analysis of Hedges' g effect-size of all RCTs comparing the efficacy of disulfiram and controls.

When combining the 22 RCTs, our meta-analysis showed a significant success rate of disulfiram compared to controls: g = .58 (95%CI = .35–.82) (Figure 2). *I^2^* was 72%. A visual inspection of the funnel plot revealed asymmetry, indicating a possible publication bias. The trim-and-fill analysis indicated that there were 4 potentially missing studies on the left side of the funnel plot. Nevertheless, the summary effect-size remained significant after correcting for the putatively missing studies [adjusted effect-size g = .47 (95%CI = .24–.71)]. The summary effect-size reached significance in all cases in the leave-one-out analysis, with summary effect-sizes varying from g = .53 to g = .63 (all p<.001). Meta-regression indicated a significant effect of publication year (β = .03, p<.001) and treatment duration (β = −.01, p<.001), but not of disulfiram dosage or risk of bias score.

**Figure 3:**
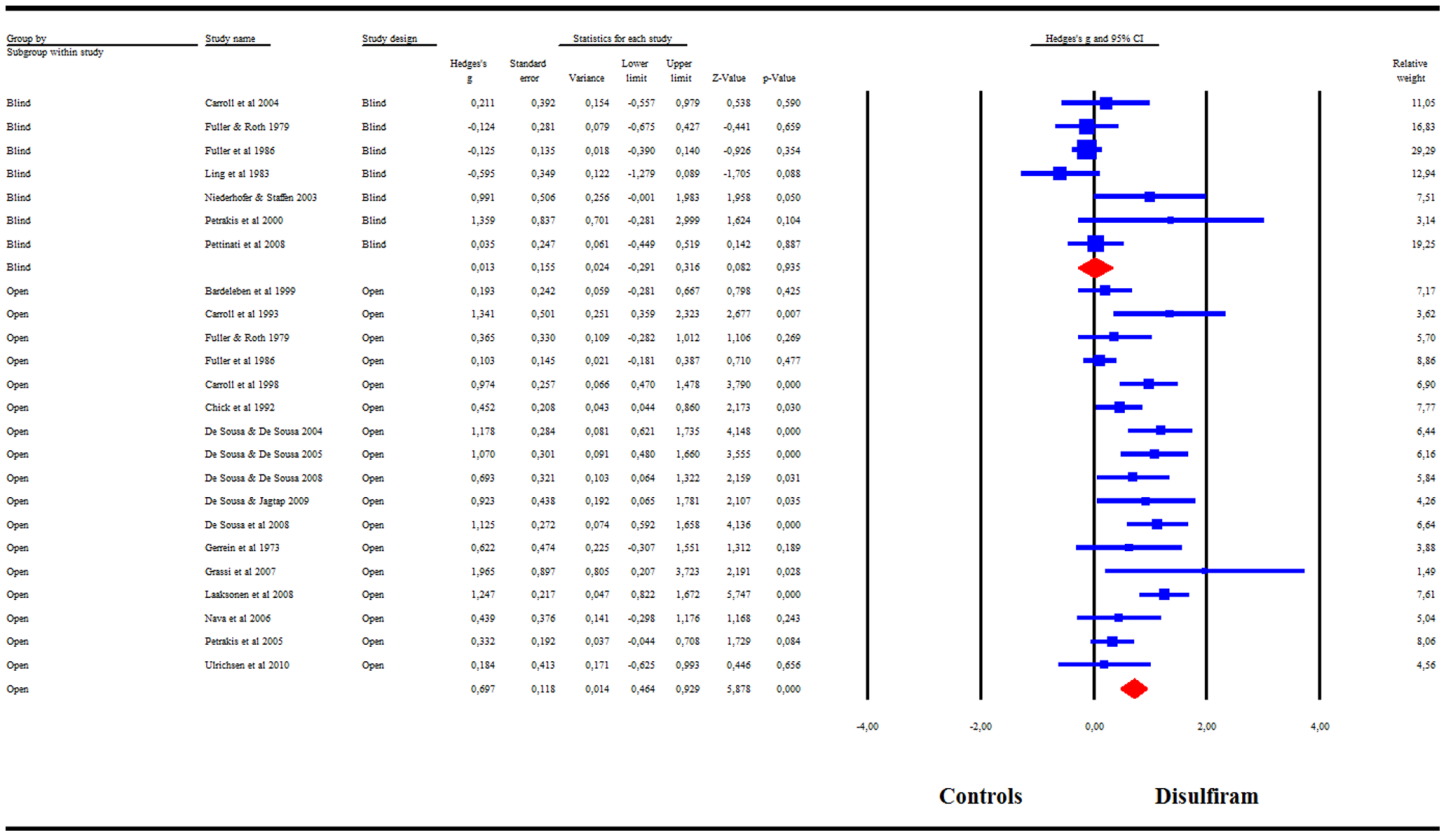
Meta-analysis for blinded versus open-label RCTs. Meta-analysis of Hedges' g effect-size comparing the efficacy of disulfiram and controls in blinded versus open-label RCTs.

The subgroup analysis comparing blind and open-label RCTs indicated that only the open-label trials showed a significant superiority of disulfiram over controls: g = .70 (95%CI = .46–.93), whereas the RCTs with blind designs showed no efficacy of disulfiram as compared to controls: g = .01 (95%CI = .−.29–.32) (Figure 3). *I^2^* = 65% for open-label studies and *I^2^* = 43% for blind studies. Having validated our hypothesis that a blind design is unsuitable for assessing disulfiram efficacy, we excluded the blind trials from the subsequent analyses. A visual inspection of the funnel plot for open-label studies revealed asymmetry, indicating possible publication bias. The trim-and-fill analysis indicated that there were 2 potentially missing studies on the left side of the funnel plot. Nevertheless, the summary effect-size remained significant after correcting for the supposedly missing study (adjusted effect-size g = .65 (95%CI = .42–88). The summary effect-size reached significance in all cases in the leave-one-out analysis, with summary effect-sizes varying from g = .64 to g = .75 (all p<.001). Meta-regression indicated no significant effect of publication year, disulfiram dosage, treatment duration, or risk of bias score.

**Figure 4:**
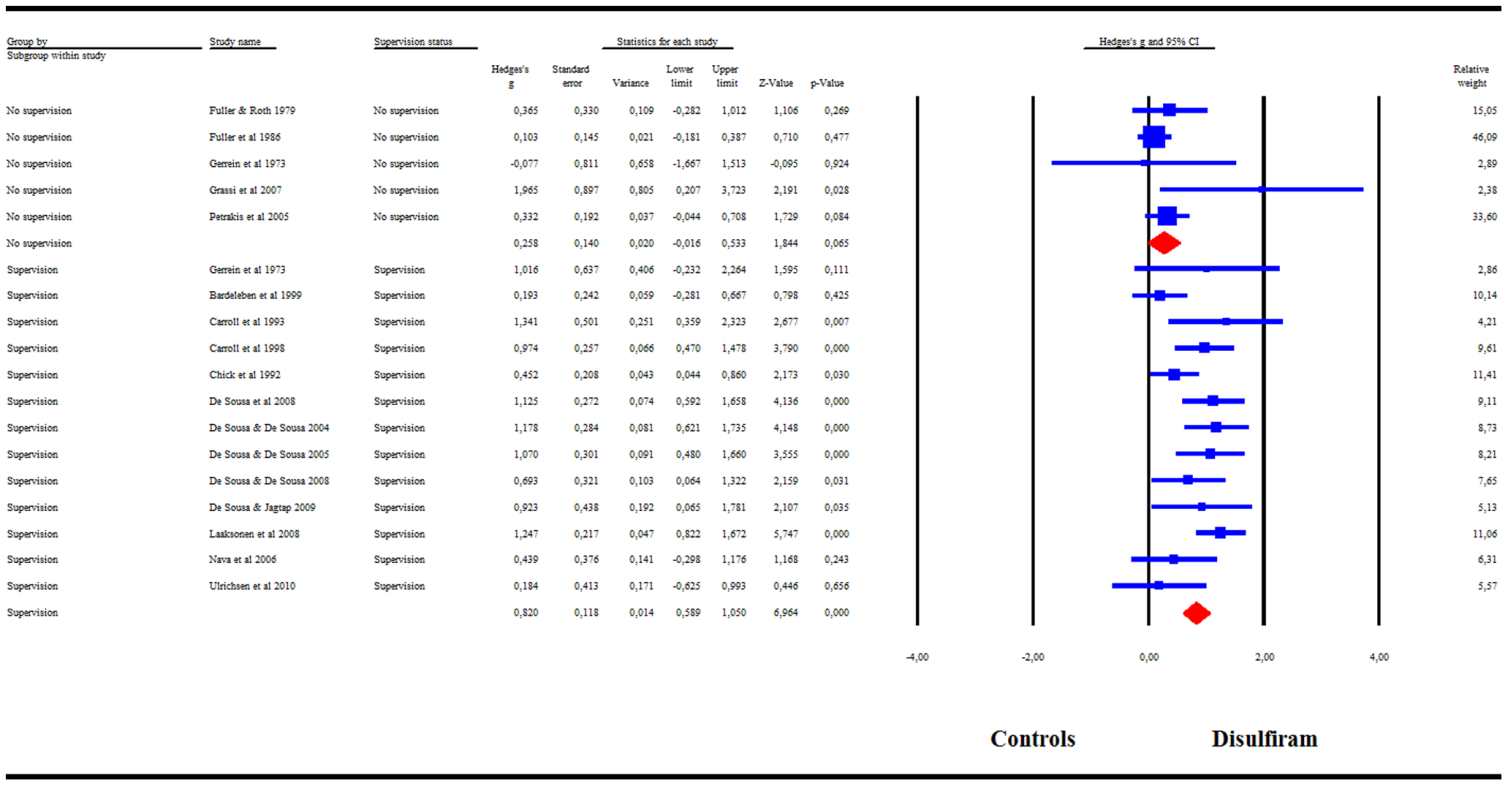
Meta-analysis of RCTs with supervision versus no supervision. Meta-analysis of Hedges' g effect-size comparing the efficacy of disulfiram and controls in RCTs with supervision versus no supervision.

The subgroup analysis by supervision categories showed disulfiram to be significantly superior to the control condition when medication compliance was supervised: g = .82, 95%CI = .59–1.05 (Figure 4). *I^2^* was 46%. When disulfiram treatment was not supervised, however, the treatment showed no significant efficacy as the results fell short of the significance level: g = .26 (95%CI = −.02–.53). In addition these two categories of studies were significantly different from each other. A visual inspection of the funnel plot for supervised studies revealed no asymmetry, indicating no publication bias. The summary effect-size reached significance in all cases in the leave-one-out analysis, with summary effect-sizes varying from g = .74 to g = .89 (all p<.001). Meta-regression indicated no significant effect of treatment duration, publication year, disulfiram dosage, or risk of bias score.

**Figure 5:**
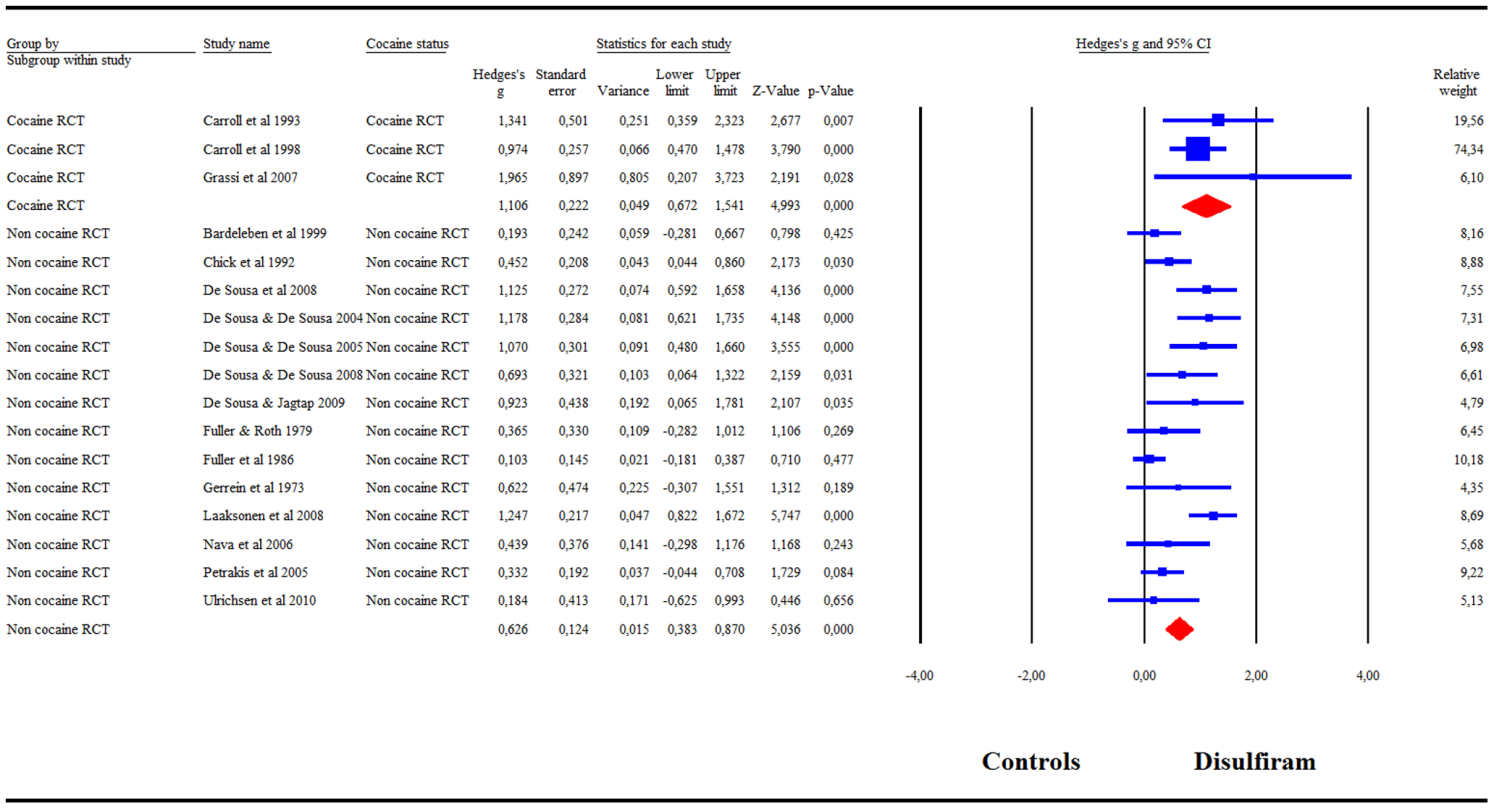
Meta-analysis of RCTs that included cocaine versus non cocaine subjects. Meta-analysis of Hedges' g effect-size comparing the efficacy of disulfiram and controls in RCTs that included alcohol dependent cocaine subjects versus those that did not include cocaine subjects.

The subgroup analysis by cocaine categories showed a significant disulfiram efficacy as compared to the control condition in both cocaine studies (g = 1.11, 95%CI = .67–1.54) and non cocaine studies primarily enrolling alcoholics (g = .63, 95%CI = .38–.87) (Figure 5). In cocaine studies, *I^2^* was 0%. In non cocaine studies, *I^2^* was 66%.

**Figure 6:**
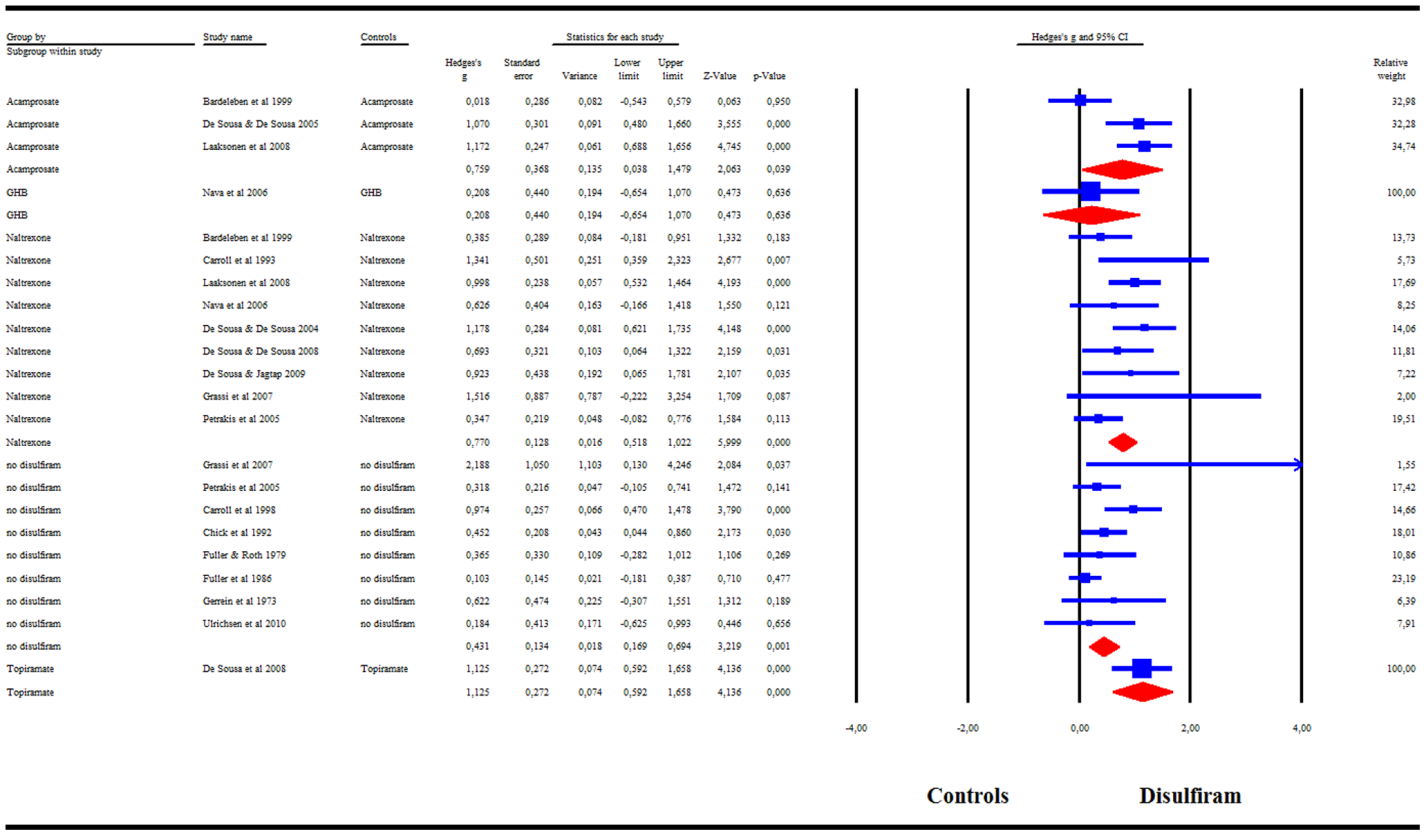
Subgroup analysis of Hedges' g effect-size comparing the efficacy of disulfiram and controls by control types.

The subgroup analysis by control condition categories showed a significant disulfiram superiority as compared to naltrexone (g = .77, 95%CI = .52–1.02), acamprosate (g = .76, 95%CI = .04–1.48), and to the no disulfiram condition (g = .43, 95%CI = .17–.69) (Figure 6). Our meta-analysis showed that disulfiram was also more effective than topiramate, and no different from GHB, but as only one study could be included for each of two comparisons, our meta-analysis does not add valuable information to this question. Heterogeneity measures were *I^2^* = 81% for the acamprosate controlled studies; *I^2^* = 26% for the naltrexone controlled studies; and *I^2^* = 44% for the no disulfiram controlled studies.

### The Safety of Disulfiram

**Figure 7:**
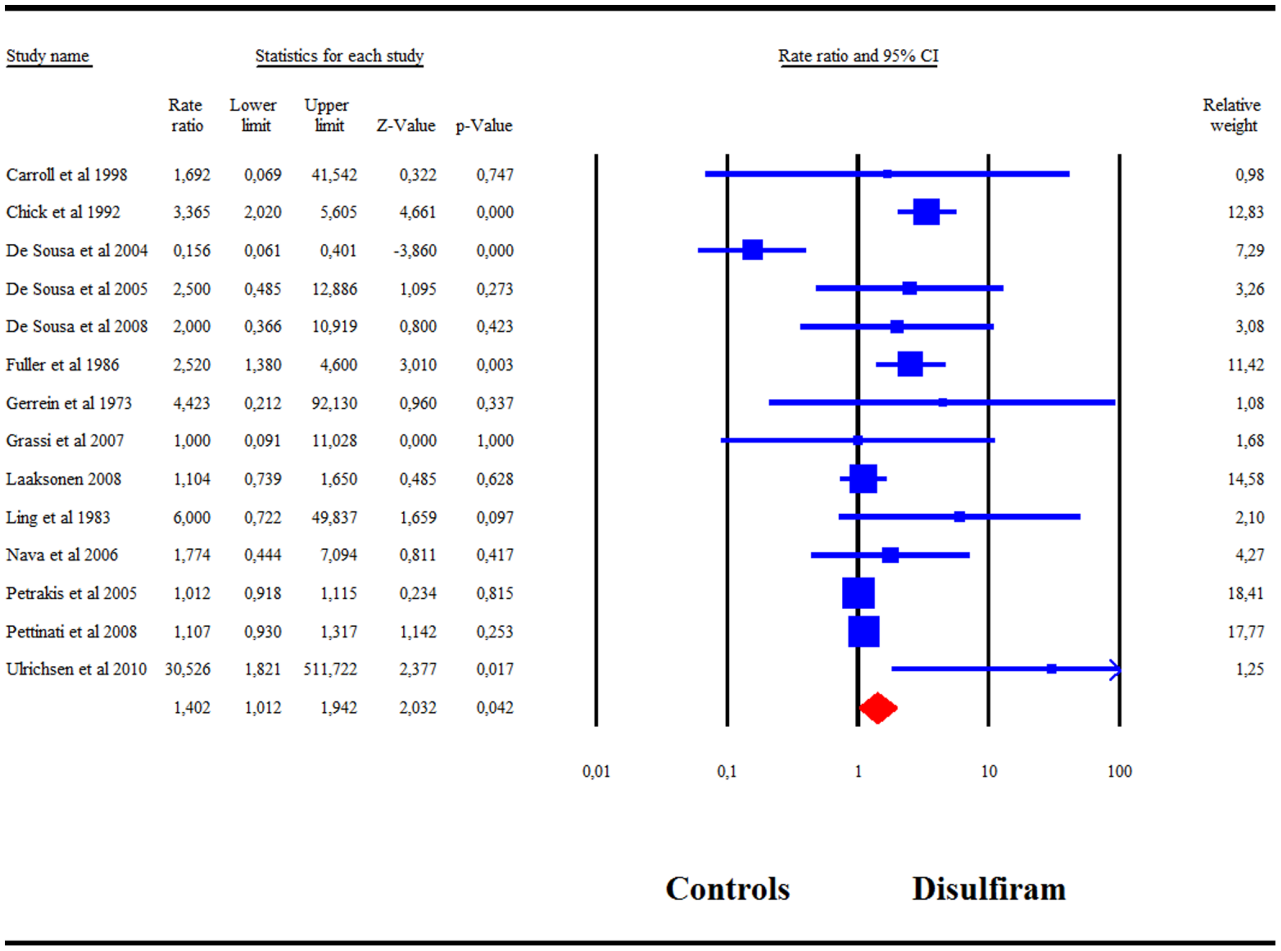
Meta-analysis of adverse events rate ratio comparisons in controls and disulfiram treated patients.

Figure 7 shows the adverse events rate ratio comparisons in disulfiram treated patients and controls. Disulfiram was associated with an increased risk of any adverse events compared with controls: adverse events rate ratio  = 1.40 (95%CI 1.01–1.94). Adverse events were reported in 73% of the studies. In the combined disulfiram groups reporting adverse events (n = 962), eight participants reported serious adverse events requiring hospitalization. Of those, two were hospitalized less than one day and then immediately resumed their participation [25]. The other six were from one other study, but according to the authors, three of them returned to complete the study [38]. In the combined control groups, six participants reported serious adverse events requiring hospitalization from three studies [38], [39], [42]. In one cocaine study, serious adverse events were not specified for those patients with a co-dependency alcohol and cocaine [14].

A total of 13 deaths were reported: one from the combined disulfiram groups [40], six from the control groups [38], [40], and six unspecified. Laaksonen et al and Petrakis et al stated that these deaths were not related to their studies. In one large study (n = 605), six deaths were reported, but the authors did not specify if these deaths were from the disulfiram group or the control group [27].

## Discussion

Our meta-analysis clearly showed significant efficacy of disulfiram on our primary endpoint that was effect-size. Our first hypothesis that disulfiram should be effective compared to controls only in open-label rather than blinded RCTs was confirmed. In open-label trials, disulfiram was more effective than controls, while there was no difference in the efficacy of disulfiram compared to placebo in blinded RCTs. The effect of disulfiram on maintaining abstinence or preventing relapse is mediated by DBH and/or ALDH inhibition as has been proposed in studies mentioned previously on cocaine dependence and pathological gambling. It is noteworthy that our results in double-blind, placebo controlled trials do not advocate for a direct pharmacological effect of disulfiram in preventing alcohol relapse.

In the field of pharmacology, the use of double-blind studies is the gold standard for determining efficacy because blinded trials reduce the influence of outside factors and biases. They help to ensure that all groups are equally matched in terms of possible biases, thus leveling the playing field for all participants. Double-blind studies lower the risk of bias such as social desirability, social support, and perceptions of participants as well as medical and research personnel involved in the study. In the case of disulfiram, the objective of the drug is to prevent consumption of alcohol by psychologically creating an expectancy of being sick if alcohol is consumed. The drug's effectiveness depends directly upon the patient's anticipations. Because the action on potential drinking behavior depends upon a thought process, disulfiram can be considered a pharmacologically assisted psychotherapy. Because of this similarity to psychotherapy, and because psychotherapy studies are necessarily open, only open-label studies could show efficacy in disulfiram RCTs.

Blinded designs for disulfiram research prevent the evaluation of the crucial aspect differentiating the disulfiram and control groups, the psychological threat. Blinding distributes the threat evenly amongst the arms of a study, whereas open-label trials allow the psychological threat to be present in only the disulfiram arm compared to controls. For this reason, we did not include blinded RCTs in our meta-analyses for the subgroups supervision, cocaine, and various controls.

Two of the blinded studies included an open-label and blind design, an admirable originality [27], [39]. In the blind portion, while both groups were informed that they were being given disulfiram, the authors dissimulated the dose: one group received 250 mg and the other received 1 mg, a pharmacologically ineffective dose. In the blind portion, as could be expected, no significant difference between disulfiram and the control groups was found. But surprisingly, even in the open-label portion comparing disulfiram to no disulfiram, there were no differences between the groups. Compliance problems rendered the results quasi meaningless. In the larger study (n = 605) [27], only 20% of the 577 who completed the study were compliant. It is noteworthy that a subset of cooperative drinkers reported significantly fewer drinking days when given disulfiram. Supervision might have made a substantial difference in the results of this study and had an important effect on our meta-analysis, as this study alone comprised 26% of the cumulative total of subjects.

Historically, based on this and other blinded trials, disulfiram developed a bad reputation as its detractors perhaps too hastily concluded that disulfiram was not any more effective than controls. But as we have shown, blinded experiments serve no purpose in their attempt to evaluate disulfiram efficacy. Control groups must not be led to believe that they have taken disulfiram so that differences in expectancies appear. Knowledge of disulfiram and its consequences are essential to its function. Disulfiram helps patients learn this new non drinking behavior, this ability to exercise self-control. As described by Brewer and Streel (2003), refraining from alcohol consumption is a learning process and requires intentionally becoming alcohol intolerant through exposure and response prevention [45]. In order to permit the learning phase to proceed despite various temptations, compliance was essential, hence the importance of supervision.

Our second hypothesis was that disulfiram should be more effective in supervised compared to unsupervised studies. We were able to confirm our hypothesis because the difference between the supervised and the unsupervised studies was significant. Our meta-analysis showed that when supervised, disulfiram performed significantly better than controls, while in unsupervised studies, disulfiram was not superior to controls on the main outcome. A closer examination of the unsupervised studies showed that in four out of the five RCTs, disulfiram was not better than the controls. Only in a small pilot study (n = 12) was there a positive disulfiram result against controls [13].

Compliance is a crucial issue in pharmacotherapy in general, but with disulfiram in particular it has been emphasized by many authors [2], [17], [18], [23]. The highest success rates have been with patients who have chosen this type of treatment and are thus highly compliant or are receiving disulfiram under supervision [46]. Some authors have gone so far as to state that unsupervised administration is of limited utility [17], [47], [48].

This raises the question of bias in supervised studies. Partnership as well as close professional supervision create high functional social support (FSS) [49]. It has been shown that FSS can have a positive effect on the proportion of days abstinent [50] and can predict treatment retention and reduction of alcohol intake [51]. A recent study has shown that FSS is associated with a higher cumulative abstinence [49]. It is probable that the positive influence of strong support had an important impact on compliance and contributed to reducing the use of alcohol. This might explain why supervised treatments had a superior success rate compared to unsupervised treatments. Nevertheless, when bias was eliminated by looking only at supervised studies in which both control and disulfiram arms were supervised, eight out of ten showed a superior performance of disulfiram compared to controls. There seemed to be no bias of social support on the superior performance of disulfiram compared to controls amongst supervised studies in which both arms were supervised.

In two supervised studies [22], [42], disulfiram was not different from controls. In the Ulrichsen et al study, the authors attributed this to a small sample size in conjunction with a probable bias of selection. Of the 158 patients who refused to participate, 63 of them refused because they wanted to be treated with disulfiram. This may have resulted in a less than average level of motivation in the sample. In addition, 67% and 41% of the control (no disulfiram) and disulfiram groups, respectively, completed the cognitive behavior therapy sessions, suggesting lower motivation in the disulfiram group. In the Bardeleben et al study, while the number of abstinent days was the same for the three groups, the time to first drink was significantly longer in the disulfiram group compared to the naltrexone and acamprosate groups.

Disulfiram unexpectedly embarked upon a new career in the cocaine dependence treatment field [52]. Most of the trials testing the efficacy of disulfiram in cocaine addicts enrolled subjects with comorbid alcohol and cocaine abuse or dependence, suggesting that the effect of disulfiram on alcohol would have a positive effect on cocaine use. Our decision to include this population is based on the high rates of comorbid alcohol/cocaine use. Numerous studies have shown that the majority of patients who abuse cocaine also abuse alcohol, ranging from 50% to 90% [53], [54], [55], [56], [57], [58]. This can be explained by the effect of cocaethylene, a pharmacologically active metabolite that is produced when cocaine and alcohol are used together that enhances and prolongs cocaine euphoria [59]. Our meta-analysis showed that treating these subjects with disulfiram for their cocaine problem resulted in a significant improvement of their alcohol condition. Disulfiram also showed its efficacy as compared to a control condition in the studies primarily enrolling alcoholics. The cocaine studies comprised 152 subjects from three studies. We did not include the results of three other cocaine studies because of their blind design [10], [14], [26].

The subgroup condition by control group categories showed efficacy of disulfiram when compared to (1) the no disulfiram condition (eight studies), (2) naltrexone (nine studies), and (3) acamprosate (three studies). These three studies had a combined total of 403 subjects. Other evidence outside of this meta-analysis in favor of disulfiram compared to acamprosate comes from retrospective data from 2002–2007 with 353 alcoholic patients [60]. In the present study, disulfiram was also superior to topiramate and showed no difference with GHB in these single trial categories, thus providing less useful information as only one study was available. The global picture of this subgroup analysis suggests a favorable image of the efficacy of disulfiram when compared with two of the most evidence based drugs for alcohol dependence.

In sum, the effect-size of disulfiram efficacy compared to various controls can be interpreted as medium (g = .70) when combining all open-label studies, or large (g = .82) when combining only studies in which compliance was supervised [28].

### Safety and Tolerance

Our meta-analysis of the safety and tolerance of disulfiram showed that there was no difference between the disulfiram and control groups in studies reporting deaths and serious adverse events requiring hospitalization. There were, however, significantly more adverse events reported for disulfiram than for controls as shown in Figure 7.

Disulfiram appears to be a safe medication in carefully screened populations. Indeed, as pointed out by Brewer, “compared with the toxicity of alcohol, the toxicity of disulfiram is trivial” [61]. The safety of disulfiram can be attributed primarily to the selection of subjects in RCTs, for the screening process is generally more rigorous than that used for clinical disulfiram use. In a recent systematic review of case reports and clinical trials using disulfiram for alcohol and/or cocaine use or dependence [62], the authors concluded that disulfiram has an acceptable risk profile and is generally safe when used according to the recommendations. They noted that case reports consisted of dermatological, neurological, psychiatric, hepatic, and cardiac adverse events as well as drug-drug interactions and neuroimaging findings. Other authors noted the more common problems of skin rash, halitosis, and fatigue [63].

Iber et al studied liver toxicity in the Fuller et al (1986) study by analyzing the changes in liver status of 605 alcoholics [64]. They found that in those with a liver anomaly, the majority were drinking, concluding that the modest changes that occurred in their liver tests were more related to drinking than to the disulfiram they were taking.

In alcoholics who also abused cocaine, the side effects reported were similar to those reported with the alcoholic only population [65]. In their review of the safety of disulfiram in randomized clinical trials, the authors concluded that use of disulfiram was effective and safe because of adequate medical monitoring. This included attention to comorbid disorders, drug interactions, appropriate dosage, supervision, and clear patient instructions.

### Limits

Most subjects in the meta-analysis were men (89%). This should be kept in mind in interpreting these results for women. In three studies, we included subjects who received disulfiram along with another treatment. In one of these trials, the additional treatment was a placebo [38]. In the two others using methadone treated subjects, the disulfiram (given to the experimental group) and the placebo (given to the control group) were placed in the methadone [14] or given with the methadone [32] for compliance purposes. We chose not to analyze any comparisons in which disulfiram was combined with naltrexone or similar abstinence supportive drugs. In addition, we intentionally excluded studies in which all experimental groups received the same dose of disulfiram so as to evaluate other aspects of treatment (i.e., the effects of supervision or behavior therapy) [1].

Limits exist in supervised studies for compliance is often based on self-reports. In the five Indian studies [24], [33], [35], [36], [37], family members were asked to supervise, but there was no real method to verify this supervision. The authors noted that India has a good social support system. Negative reinforcement may have helped compliance also as participants were told that they would be excluded for non-compliance.

Studies of disulfiram are heterogeneous. Trial methodologies were highly diverse because since its discovery over 60 years ago no consensus has been reached as to trial methodology. Thus the heterogeneity of the studies was unavoidable, characterized by a substantial I-square. The only subgroups of studies that did not display a high level of heterogeneity were those on cocaine and naltrexone.

As shown by the meta-regressions, the high heterogeneity was partially explained by the wide range of publication years and treatment durations. Notably, heterogeneity was found to be low when disulfiram was compared with each control condition independently, suggesting that the important number of control conditions might explain a substantial amount of heterogeneity.

According to the guidelines of the *Cochrane Handbook for Systematic Reviews of Interventions* [29], a lack of blinding of participants and personnel in randomized trials increases the risk of bias. The areas most affected are performance (i.e., there may be differences in the care that is provided in each arm) and detection bias (i.e., there may be differences between the groups in how outcome is determined). For many reasons discussed previously, while double-blinding is the standard method for medication trials in general, it is unsuitable for disulfiram trials. One might infer that this meta-analysis is biased by using predominantly open-label trials. In disulfiram research, we propose that one revisit the meaning of quality RCTs. A meta-analysis of disulfiram would be of no practical use if it consisted of only double-blind trials. The use of an open-label design in efficacy trials with this atypical medication could be viewed as indispensable rather than as evidence of poor study quality.

## Conclusion

The present work focuses on open-label trials to correct the dilutive and misleading effect that blinded trials have had on the question of disulfiram efficacy. In formulating the hypothesis that only open trials can determine disulfiram efficacy, this meta-analysis addresses a cardinal methodological flaw that was not considered in a previous meta-analysis. Disulfiram was shown to be more effective than controls in supervised than non supervised RCTs. Based on a larger sample than a previous meta-analysis [19], adding eleven studies (887 subjects), it broadens the evaluation of disulfiram efficacy to include alcoholics with concomitant cocaine abuse or dependence. In addition, it offers a meta-analytic evaluation of the safety of disulfiram, a poignant issue to this day.

In summary, how does disulfiram measure up when compared to controls in helping the alcohol dependent stay abstinent or at least relapse free? Overall, this meta-analysis demonstrated evidence in open-label trials of disulfiram efficacy compared to controls in maintaining abstinence or preventing relapse. No efficacy was revealed in blind trials. In terms of safety, there was no difference between the disulfiram and control groups in studies reporting deaths and serious adverse events requiring hospitalization. Adverse events, however, were reported more for disulfiram than for controls. In spite of the limitations mentioned above, our meta-analysis allowed us to draw strong conclusions about the efficacy of disulfiram compared to other abstinence supportive pharmaceutical treatments or to no disulfiram in open-label, supervised studies for problems of alcohol abuse or dependence.

## Supporting Information

Checklist S1 PRISMA checklist.
(DOC)

Search Strategies S1 (DOC)
