# Supplementary material for: Disulfiram Efficacy in the Treatment of Alcohol Dependence: A Meta-Analysis
Source: PLoS One. 2014 Feb 10;9(2):e87366. doi: 10.1371/journal.pone.0087366 (PMC3919718; doi:10.1371/journal.pone.0087366)
Supplement: Search Strategies S1 — (DOC) [file pone.0087366.s002.doc]

S 1– Search Strategies

The initial search was conducted on the PubMed database, using the following search strategy: ((((single blind method[MeSH Terms]) OR (double blind method[MeSH Terms]) OR (random allocation[MeSH Terms]) OR ("randomized controlled trials as topic"[MeSH Terms]) OR (controlled clinical trial[Publication Type]) OR (randomized controlled trial[Publication Type])) NOT ((animal[MeSH Terms]) NOT human[MeSH Terms])) OR ((((clinical trial[Publication Type]) OR (exp CLINICAL TRIALS)) NOT ((animal[MeSH Terms]) NOT human[MeSH Terms])) NOT (((single blind method[MeSH Terms]) OR (double blind method[MeSH Terms]) OR (random allocation[MeSH Terms]) OR ("randomized controlled trials as topic"[MeSH Terms]) OR (controlled clinical trial[Publication Type]) OR (randomized controlled trial[Publication Type])) NOT ((animal[MeSH Terms]) NOT human[MeSH Terms]))) OR (( (exp Evaluation studies) OR ("follow up studies"[MeSH Major Topic]) OR ("prospective studies"[MeSH Major Topic]) OR ((control$ OR prospectiv$ OR volonteer$) AND Title/Abstract)) NOT ((((single blind method[MeSH Terms]) OR (double blind method[MeSH Terms]) OR (random allocation[MeSH Terms]) OR ("randomized controlled trials as topic"[MeSH Terms]) OR (controlled clinical trial[Publication Type]) OR (randomized controlled trial[Publication Type])) NOT ((animal[MeSH Terms]) NOT human[MeSH Terms])) OR ((((clinical trial[Publication Type]) OR (exp CLINICAL TRIALS)) NOT ((animal[MeSH Terms]) NOT human[MeSH Terms])) NOT (((single blind method[MeSH Terms]) OR (double blind method[MeSH Terms]) OR (random allocation[MeSH Terms]) OR ("randomized controlled trials as topic"[MeSH Terms]) OR (controlled clinical trial[Publication Type]) OR (randomized controlled trial[Publication Type])) NOT ((animal[MeSH Terms]) NOT human[MeSH Terms])))))) AND ((exp Disulfiram/) OR (disulfiram) OR (disulfirame)).

A second search was conducted on the EMBASE database using the following search strategy: ‘disulfiram'/exp OR disulfiram AND 'alcohol'/de AND 'alcoholism'/de AND 'human'/de AND 'controlled study'/de AND 'article'/it.

A final search was conducted the Cochrane Central Register database using the key words alcohol, disulfiram and trials.
